# Mutations in RNA Methyltransferase Gene *NSUN5* Confer High Risk of Outflow Tract Malformation

**DOI:** 10.3389/fcell.2021.623394

**Published:** 2021-04-21

**Authors:** Yifeng Wang, Tao Jiang, Jiani Xu, Yayun Gu, Yan Zhou, Yuan Lin, Yifei Wu, Wei Li, Cheng Wang, Bin Shen, Xuming Mo, Xiaowei Wang, Bin Zhou, Chenyue Ding, Zhibin Hu

**Affiliations:** ^1^State Key Laboratory of Reproductive Medicine, Nanjing Medical University, Nanjing, China; ^2^Department of Epidemiology and Biostatistics, Center for Global Health, Nanjing Medical University, Nanjing, China; ^3^Department of Cardiothoracic Surgery, Children’s Hospital of Nanjing Medical University, Nanjing, China; ^4^Department of Thoracic and Cardiovascular Surgery, The First Affiliated Hospital of Nanjing Medical University, Nanjing, China; ^5^Department of Genetics, Albert Einstein College of Medicine, New York, NY, United States; ^6^Center of Reproduction and Genetics, The Affiliated Suzhou Hospital of Nanjing Medical University, Suzhou Municipal Hospital, Gusu School, Nanjing Medical University, Suzhou, China

**Keywords:** *NSUN5*, tetralogy of Fallot, 5-methylcytosine, outflow tract (OFT), heart development

## Abstract

*NSUN5*, encoding a cytosine-5 RNA methyltransferase and located in the 7q11.23 locus, is a candidate gene for tetralogy of Fallot (TOF). Deletion of the 7q11.23 locus in humans is linked to cardiac outflow tract (OFT) disorders including TOF. We identified four potential pathogenic mutations in the coding region of *NSUN5* and which were enriched in TOF patients by an association study of 132 TOF patients and 2,000 in-house controls (*P* = 1.44 × 10^–5^). We then generated a *Nsun5* null (*Nsun5*^–/–^) mouse model to validate the human findings by defining the functions of *Nsun5* in OFT morphogenesis. The OFT did not develop properly in the *Nsun5* deletion embryonic heart. We found a misalignment of the aorta and septum defects caused by the delayed fusion of the membraneous ventricular spetum as an OFT development delay. This caused OFT development delay in 27 of 64 (42.2%) *Nsun5*^–/–^ mice. Moreover, we also found OFT development delay in 8 of 51 (15.7%) *Nsun5*^+/–^ mice. Further functional experiments showed that the loss of *Nsun5* function impaired the 5-methylcytosine (m^5^C) modification and translation efficiency of essential cardiac genes. *Nsun5* is required for normal OFT morphogenesis and it regulates the m^5^C modification of essential cardiac genes. Our findings suggest the involvement of *NSUN5* in the pathogenesis of TOF.

## Introduction

Congenital heart disease (CHD) is the most common congenital anomaly in live births (van der Bom et al., 2011). Approximately one-third of CHD cases affect the cardiac outflow tract (OFT), and congenital OFT malformations have significantly high morbidity and mortality rates in children and adults (Go et al., 2014). Tetralogy of Fallot (TOF) is a severe syndrome of complex OFT abnormalities including overriding aorta, pulmonary artery stenosis, ventricular septal defects, and right ventricular hypertrophy (MIM #187500) (Go et al., 2014). TOF occurs in 1:3,000 live births worldwide (van der Linde et al., 2011). Despite improvements in surgical repair, the long-term outcome of TOF remains a huge health problem due to right ventricular dyssynchrony and sudden cardiac death (Valdeomillos et al., 2019).

The deletion of the 7q11.23 locus was reported to be associated with TOF (Pober, 2010; Yuan, 2017). A typical 7q11.23 deletion contains 25 genes that encode transcriptional regulators, signaling molecules, and other factors that function in various cellular processes (Francke, 1999). However, the essential gene that causes TOF has not been identified to date. A recent study reported that developmental disorders were caused by a 7q11.23 deletion that included several genes with roles in epigenetic regulation, including methylation (Strong et al., 2015). The *NSUN5* gene, a member of the NOL1/Nop2/sun protein family, is one of the 25 genes in the deleted 7q11.23 locus (Schubert, 2009; Sharma et al., 2013). *NSUN5* encodes a cytosine-5 RNA methyltransferase that is required for RNA 5-methylcytosine (m^5^C) modification (Schosserer et al., 2015). m^5^C is an abundant post-transcriptional epigenetic modification (Squires et al., 2012; Amort et al., 2017; Yang et al., 2017). It was reported that post-transcriptional modification is necessary for heart development. Furthermore, a recent study revealed that a marked excess of mutations in genes involved in the production, removal, or reading of *H3K4* and *H3K27* epigenetic modifications, were important for cardiac development (Zaidi et al., 2013). However, whether *Nsun5* is a candidate gene in the 7q11.23 locus deletion that results in TOF and whether its m^5^C modification has a role in the regulation of cardiac genes during OFT development are not known.

In this study, we performed direct Sanger sequencing to evaluate putative functional mutations in the *NSUN5* coding region and we modeled the OFT defects in *Nsun5* deletion mice. Our data suggest that *NSUN5* mutations are disease factors in TOF, which mediate their effects via the disrupted m^5^C modification of key genes in cardiac development.

## Materials and Methods

### Ethics Statement

This study was approved by the institutional review boards of all participating hospitals and all TOF cases and controls provided informed consent under the research protocol approved by Nanjing Medical University. All investigations conformed to the principles outlined in the Declaration of Helsinki. All animal procedures conformed to the Guidelines for the Care and Use of Laboratory Animals published by the National Institution of Health and were approved by the Institutional Animal Care and Ethical Committee of Nanjing Medical University (IACUC 1709020).

### Study Subjects

The study included 132 TOF cases from the First Affiliated Hospital of Nanjing Medical University (Nanjing, China) and the Affiliated Children’s Hospital of Nanjing Medical University (Nanjing, China). Cases with known chromosomal abnormalities were excluded. Cases with a positive first-degree relative (parent, child, or sibling) with a family history of TOF, maternal exposure to teratogens such as pesticides or therapeutic drugs, maternal diabetes mellitus, or phenylketonuria during the intrauterine period were also excluded. A diagnosis of TOF was based on echocardiography and confirmed by surgery. Controls without TOF malformations were recruited during the same period and in the same geographic area. Subjects were excluded from the control group if they had any congenital anomalies.

### DNA Extraction and Direct Sanger Sequencing

Approximately 2 ml of peripheral blood from each subject was obtained to extract genomic DNA (500 ng) and assessed by gel electrophoresis for quality. We downloaded the sequence of the *NSUN5* coding region from the human reference genome (GRCh37/hg19). We used online software Primer 3 (v.0.4.0) to design primers. The *NSUN5* coding region was amplified by PCR with 10 primer pairs. PCR conditions were as follows: denaturation at 95°C for 5 min, 35 cycles (95°C for 30 s, 60°C for 30 s, 72°C for 2 min), extension at 72°C for 5 min, and then holding at 4°C. After the PCR reaction, products were sent for direct Sanger sequencing (Tsingke, Nanjing). The sequencing data were visualized by Sequencing Analysis (Applied Biosystems), and variants were analyzed using the online software NCBI Basic Local Alignment Search Tool (BLAST). The predicted functional mutations were annotated using the Combined Annotation Dependent Depletion (CADD) tool. All primer sequences for Sanger sequencing are listed in Supplementary Table 1.

### Cell Culture and Transfection

HEK293T cells were cultured in DMEM (Gibco) supplemented with 10% fetal bovine serum (Gibco), 100 U/ml penicillin, 100 μg/ml streptomycin and grown at 37°C in a humidified atmosphere with 5% CO_2_. The wild-type and mutant forms of the *NSUN5* coding sequence were cloned into a pcDNA3.1 + C-HA plasmid and then transfected into cells using Lipofectamine 2000 (Thermo Fisher) according to the manufacturer’s instructions.

### RNA Extraction, Reverse-Transcription, and Quantitative Real-Time PCR Assay

Total RNA was extracted from cells by TRIzol LS Reagent (Invitrogen). Then, 1,000 ng good quality RNA was reverse transcribed to complementary DNA (cDNA) using the PrimeScript^TM^ RT Master Mix (Takara). All cDNA sample runs were performed in triplicate using the SYBR PCR Master Mix reagent kit (Takara) and quantitative real-time PCR was performed on an ABI Q7 real-time PCR System (Applied Biosystems). Endogenous expression of *GAPDH* was used to normalize expression levels. The primer sequences are listed in Supplementary Table 2.

### Generation of *Nsun5*^–/–^ Mice

Mice were maintained at a temperature of 23 ± 2°C, humidity of 55 ± 5%, and a 12:12 h light/dark cycle in the Animal Research Center of Nanjing Medical University with access to food and water. The *Nsun5*^–/–^ mouse was generated by the CRISPR/Cas9 genome editing system. Single guide RNAs (sgRNAs) were designed to recognize Exon 3 of *Nsun5*. Two oligos were annealed and cloned into a pUC57-sgRNA expression vector (Addgene 51132) to generate sgRNA plasmids. The pooled system of single-cell embryo microinjection was described previously (Shen et al., 2014). Founder mice were backcrossed to C57BL/6 mice for four generations to obtain a pure background. Genomic DNA from mouse tail biopsies was collected for genotyping by PCR amplification. No predicted off-target sites were found by PCR amplification or Sanger sequencing of 4 wild-type, 4 heterozygous, and 4 homozygous mice. All sgRNAs and primer sequences are listed in Supplementary Table 2.

### Mouse Anesthesia and Euthanasia

Pregnant mice were sacrificed by an overdose of isoflurane in a sealed container.

### Histological Analysis of Mouse Hearts

Histological analysis was performed to identify the phenotype of *Nsun5*^–/–^ mice. Hearts were isolated and fixed in 4% paraformaldehyde (PFA), dehydrated, embedded in paraffin, and sectioned frontally at 6-μm thickness. Hematoxylin and Eosin (H&E) was carried out using standard protocols. Stained sections were photographed with a Zeiss microscope and images were processed for quantitative analysis.

### Immunofluorescence Staining

Hearts were collected and fixed in 4% PFA on ice for 2 h, rinsed in PBS and dehydrated in 15 and 30% sucrose overnight at 4°C until the hearts sunk completely. Then, they were embedded in optimal cutting temperature (OCT) compound and stored at −80°C. Hearts were sectioned at 8-μm thickness on positively charged slides and stored at −20°C. For staining, hearts were washed in PBS and blocked with goat serum for 30 min at room temperature before being incubated overnight at 4°C with primary antibodies. After three washes with PBS for 5 min each, samples were stained for 1 h at room temperature with secondary antibodies followed by 4′,6-diamidino-2-phenylindole dihydrochloride (DAPI) staining for nuclei visualization. Images were acquired by a confocal laser scanning microscope (ECLIPSE-Ti, Nikon) and processed for quantitative analysis. The antibodies were as follows: cTnT (Abcam, ab8295; 1:100 dilution), Ki67 (GB1303030-2, 1:200 dilution), and Tpm1 (Abcam, ab55915; 1:100 dilution).

### m^5^C Dot Blot Assay

Total tissue RNA was extracted using the TRIzol LS Reagent (Invitrogen) according to the manufacturer’s protocols. mRNA was isolated from total RNA using the Dynabeads mRNA purification kit (Ambion). RNA samples were immobilized twice by UV cross-linking (Stratalinker 2400) and then incubated at 80°C for 1 h. The blots were blocked by blocking buffer (10% SDS, 1 mM EDTA, 1 × PBS) for 20 min. The mRNAs for dot blot analysis were incubated with specific m^5^C antibody (Diagenode, RD-004, 1:100 dilution) at 4°C overnight. The membrane was incubated with HRP-conjugated goat anti-mouse IgG (DakoCytomation, P0447, 1:10,000 dilution) at room temperature for 1 h. The membrane was washed four times for 10 min each with wash buffer and then incubated with ECF substrate (GE Healthcare) for 5 min in darkness at room temperature to visualize the spots. The intensity of the blot signal was calculated using ImageJ software.

### RNA Bisulfite Sequencing (BS-Seq)

For BS-seq, 2 μg of total RNA for each sample was rRNA depleted using a NEBNext^®^ rRNA Depletion Kit (New England Biolabs, United States). Then, rRNA-depleted RNA was bisulfite converted and purified using the EZ RNA methylation Kit (Zymo Research). According to the manufacturer’s instructions, RNA libraries were constructed with the TruSeq Stranded Total RNA Library Prep Kit (Illumina, United States). The library quality was evaluated with the BioAnalyzer 2100 system (Agilent Technologies, United States). Library sequencing was then performed with 150 bp paired end reads on an Illumina Hiseq instrument.

### BS-Seq Data Analysis

Paired-end reads were harvested from the Illumina HiSeq 4000 sequencer and quality controlled by Q30. After 3′ adaptor-trimming and the removal of low-quality reads using cutadapt software (v1.9.3), clean reads of BS-treated libraries were aligned to the reference genome (GRCm38/mm10) by meRanGh (one component of meRanTK) software. The methylation status of each C within the genome was extracted by meRanCall (one component of meRankTK) software and differentially methylated sites (DMSs) were identified by meRanCompare (one component of meRanTK) software. DMSs on exons of known genes were identified and Gene Ontology (GO) pathway enrichment analysis was performed based on these DMSs genes.

### RNA Sequencing (RNA-Seq)

According to the manufacturer’s instructions, 1 μg total RNA of each sample was used to remove the rRNAs using a Ribo-Zero rRNA Removal Kit (Illumina). RNA libraries were constructed with the TruSeq Stranded Total RNA Library Prep Kit (Illumina) following the manufacturer’s instructions. Libraries were controlled for quality and quantified by the BioAnalyzer 2100 system (Agilent Technologies). Ten pM libraries were denatured as single-stranded DNA molecules, captured on Illumina flow cells, amplified *in situ* as clusters and finally sequenced for 150 cycles on an Illumina HiSeq Sequencer according to the manufacturer’s instructions.

### RNA-Seq Data Analysis

Paired-end reads were harvested from the Illumina HiSeq 4000 sequencer, and quality controlled by Q30. After 3′ adaptor-trimming and the removal of low-quality reads by cutadapt software (v1.9.3), high-quality clean reads were aligned to the reference genome (GRCm38/mm10) with hisat2 software (v2.0.4). Then, guided by the Ensembl gtf gene annotation file, cuffdiff software (part of cufflinks) was used to obtain the gene level FPKM as the expression profiles of mRNA. Fold change and *P*-values were calculated based on FPKM and differentially expressed mRNA were identified. GO pathway enrichment analysis was performed based on the differentially expressed mRNAs.

### Protein Extraction and Western Blot Analysis

Heart tissues were collected and lysed using the mammalian protein extraction reagent RIPA (Beyotime). The protein lysates were centrifuged and the supernatants were quantified using the BCA protein assay kit (Beyotime). Then, the lysates were denatured at 100°C for 5 min with 4 × LDS Sample Buffer and 10 × Sample Reducing Agent (Life Technologies). For western blotting, equal amounts of total proteins (40 μg) were resolved by 10% SDS-PAGE and transferred onto polyvinylidene fluoride membranes (Millipore). The membranes were blocked and incubated with primary antibodies at 4°C overnight before incubation with HRP-conjugated secondary antibodies. Protein bands were visualized by a molecular imager (Bio-Rad). Antibodies to Nsun5 (Santa Cruz Biotechnology, sc-376147, 1:1000 dilution), Tpm1 (Abcam, ab55915, 1:400 dilution), Myl2 (Proteintech, 10906-1-AP, 1:1000 dilution), Cox4i1(Proteintech, 11242-1-AP, 1:1000 dilution), mouse anti-Gapdh (Beyotime, A0216, 1:1000 dilution), and rabbit anti-Gapdh (Beyotime, A0208, 1:1000 dilution) were used for western blot analysis.

### Mass Spectrometry (LC-MS/MS) Assay and Data Analysis

Heart tissue proteins were extracted as described above. After the elution of proteins, they were subjected to mass spectrometric analysis. LC-MS/MS experiments were performed with an LTQ linear ion trap mass spectrometer (Thermo Finnigan) equipped with a microspray source. Fold change and *P*-values were calculated based on protein label free quantitative (LFQ) intensity and differentially expressed proteins were identified. GO pathway enrichment analysis was performed based on the differentially expressed protein genes.

### Statistical Analysis

All statistical analyses were performed between groups using the Student’s *t-*test, One-way ANOVA, Pearson’s Chi-squared test, Fisher’s exact test, or hypergeometric test, as appropriate. All reported *P-*values are two-sided and a probability of 0.05 was selected for statistical significance. We used software R 3.3.2 version for all statistical analyses.

## Results

### *NSUN5* Mutations in TOF Patients

**FIGURE 1:**
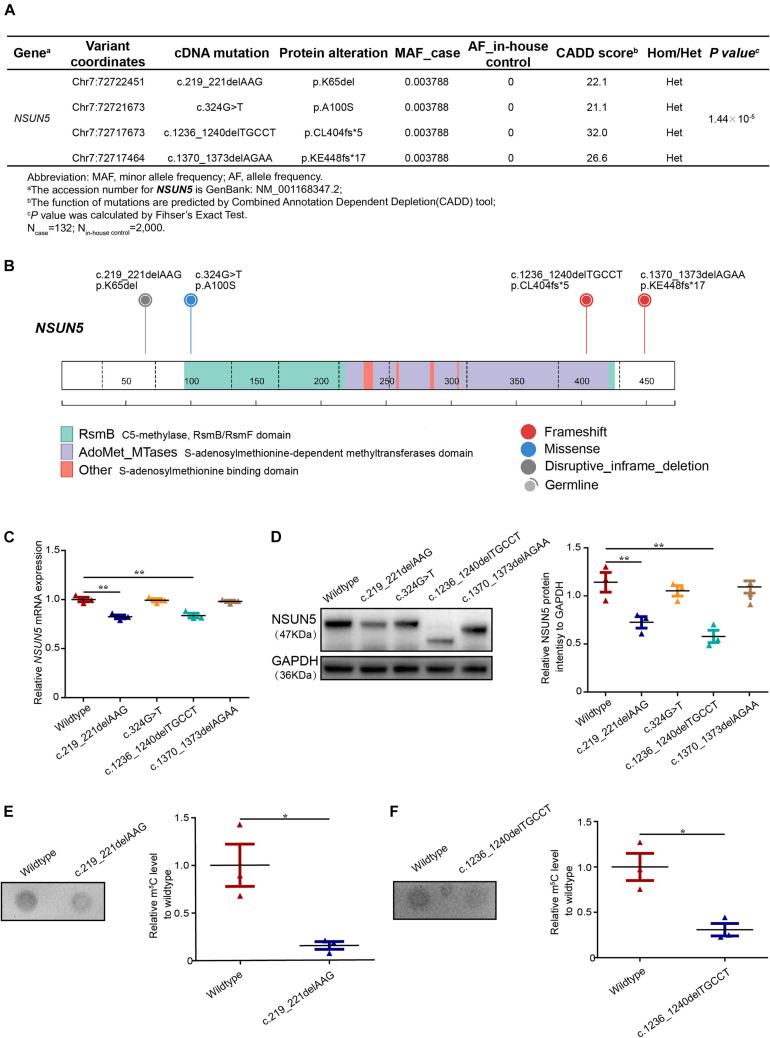
Deleterious *NSUN5* mutations in TOF patients. **(A)** Predicted deleterious mutations and association with risk of TOF (132 TOF patients and 2,000 in-house controls). **(B)** Schematic representation of a *NSUN5* structure with C5-methylase, RsmB/RsmF domain, *S*-adenosylmethionine -dependent methyltransferases domain and *S*-adenosylmethionine binding domain and the top were *NSUN5* predicted pathogenic mutations identified in our study. Fisher’s exact test was used for statistical calculation. **(C)** Quantitative real-time PCR analysis of *NSUN5* in mutant forms. **(D)** Western blot analysis of NSUN5 in mutant forms. **(E,F)** Dot blot analysis of m^5^C level in mutant forms. The values represent the mean ± SEM of three independent experiments. Unpaired Student’s *t*-test was used for statistical calculation. *P < 0.05, **P < 0.01.

We screened for potential mutations in the *NSUN5* coding region of a cohort of 132 TOF patients by Sanger sequencing. Overall, 2,000 in-house controls with no cardiac defects by whole genome sequencing were used for the screening of *NSUN5* mutations. According to the study flow shown in Supplementary Figure 1, we identified 11 mutations in TOF patients (Supplementary Table 1) and four heterozygous mutations had a Combined Annotation Dependent Depletion (CADD) score >20, which predicted a deleterious mutation (Figure 1A and Supplementary Figure 2). We combined the four mutations to evaluate the TOF risk as all these mutations were predicted to have a deleterious impact on the function of *NSUN5*. Taken together, these mutations conferred a significant risk for TOF by Fisher’s exact test (*P* = 1.44 × 10^–5^, Figure 1A). Of these, we found an in-frame mutation c.219_221delAAG (p.K65del) located in a lysine position (Figure 1B and Supplementary Figure 3A). We characterized a novel missense mutation (c.324G > T, p.A100S) in a methylase domain (Figure 1B and Supplementary Figure 3B), a non-sense mutation c.1236_1240delTGCCT (p.CL404fs^∗^5) in a *S*-adenosylmethionine-dependent methyltransferase domain (Figure 1B and Supplementary Figure 3C), and another non-sense mutation c.1370_1373delAGAA (p.KE448fs^∗^17) in a translational termination position (Figure 1B and Supplementary Figure 3D). These results suggest that *NSUN5* might be a TOF-associated gene.

### Mutant Forms Decrease *NSUN5* Expression and m^5^C Levels

To further evaluate the four *NSUN5* mutations, we conducted functional experiments of the mutant forms. We found that two mutations (c.219_221delAAG, p.K65del and c.1236_1240delTGCCT, p.CL404fs^∗^5) led to lower *NSUN5* expression by quantitative real-time PCR and western blot assays (Figures 1C,D). Moreover, dot blot assays showed that the above two mutations decreased the global m^5^C level (Figures 1E,F). These results indicate that the two functional mutations (c.219_221delAAG, p.K65del and c.1236_1240delTGCCT, p.CL404fs^∗^5) play a critical role in regulating *NSUN5* expression and m^5^C modification.

### Delayed Cardiac OFT Development in *Nsun5* Absent Mice

**FIGURE 2:**
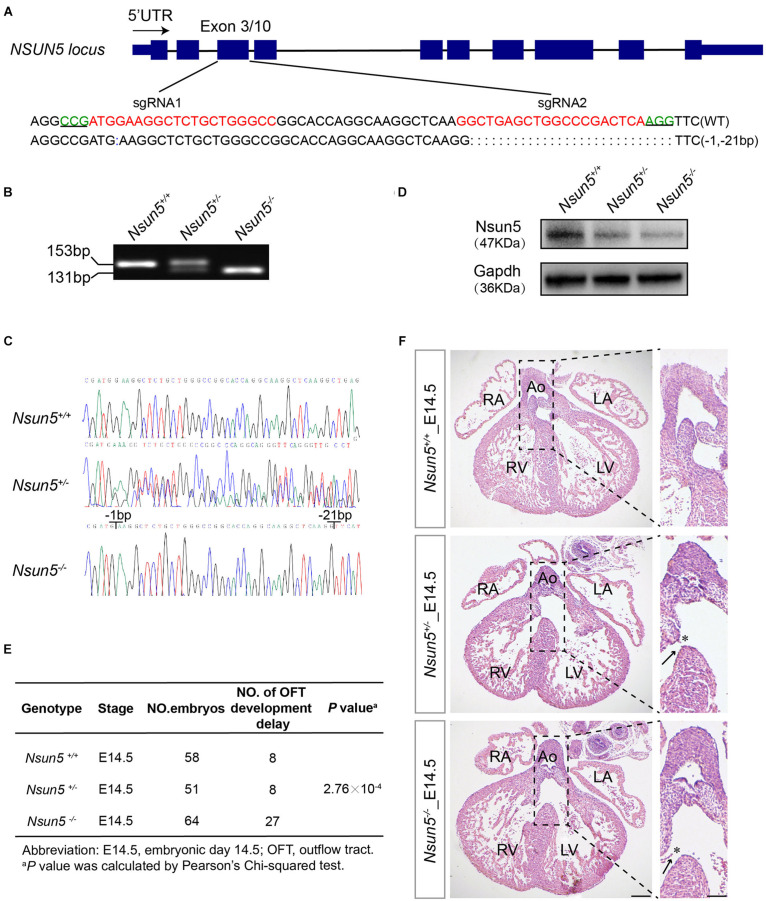
Characterization of *Nsun5* knockout mouse model and OFT septation development delay shown in *Nsun5* absent mice. **(A)** Two sgRNAs designed to target Exon3 of *Nsun5* gene. The PAM sequences were highlighted in green and the sgRNAs sequences were highlighted in red. **(B)** Genotype identification of offspring mice derived from founder mouse with 22-bp deletion. *Nsun5* DNA were from tail biopsies of wild-type (+/+), heterozygous (±) and homozygous (–/–). **(C)** Sanger sequencing verification of offspring mice. **(D)** Western blot analysis of Nsun5 in mouse heart tissue protein extracted at E14.5. **(E)** Frequency analysis of OFT development delay in E14.5 embryo hearts. **(F)** Representative H&E staining of E14.5 hearts. *Nsun5*^+/–^ and *Nsun5*^– /–^ mice E14.5 hearts clearly showed OFT aorta misalignment (asterisk) and septation defect (arrow). Scale bar = 100 or 50 μm. Pearson’s Chi-squared test was used for statistical calculation.

To study the causative effect of *Nsun5* on TOF in human patients, we generated a *Nsun5*^–/–^ mouse model using CRISPR/Cas9 (Figure 2A). The model was verified by Sanger sequencing and showed the loss of *Nsun5* protein in the embryonic day 14.5 (E14.5) *Nsun5*^+/–^ and *Nsun5*^–/–^ heart by western blot assay (Figures 2B–D). Although the heterozygous intercrosses yielded homozygous mice, we noticed an unusual transmission ratio distortion, defined as a significant departure from the expected Mendelian inheritance ratios among 153 postnatal day 1 (P1) newborns (Supplementary Figure 4). According to this observation, we inferred that the deletion may result in the death of null mice. To determine when death occurred, embryos were genotyped at E14.5 when the OFT and cardiac chamber septation are completed. The genotype ratio from E14.5 was in accordance with the expected Mendelian ratio (Supplementary Figure 4). We then examined *Nsun5*^+/–^ and *Nsun5*^–/–^ hearts at E14.5 and found that OFT did not develop properly. Of note, we found a misalignment of the aorta and septum defects caused by the delayed fusion of the membraneous ventricular spetum as an OFT development delay (Figures 2E,F). This caused OFT development delay in 27 of 64 (42.2%) *Nsun5*^–/–^ mice (Figure 2E). Moreover, we also found OFT development delay in 8 of 51 (15.7%) *Nsun5*^+/–^ mice (Figure 2E). We found different genotypes of *Nsun5* mouse hearts at E15.5 and P1. OFT developmental defects were observed at E15.5 and P1 in *Nsun5*^+/–^ (4/28, 14.3% at E15.5 and 8/60, 13.3% at P1) and *Nsun5*^–/–^ (9/36, 25% at E15.5 and 4/15, 26.7% at P1) mouse hearts (Supplementary Figures 5A–D). No OFT developmental defects were observed at E15.5 and P1 in wildtype mice hearts. The results suggest that the wildtype mice would get phenotypic recovery with advance of development process, yet part of *Nsun5* null mice still had OFT developmental delay. Nonetheless, these data demonstrate a critical role for *Nsun5* in OFT development because mice with a loss of *Nsun5* had a high risk of developing OFT malformations.

### Decreased Cell Proliferation in *Nsun5* Absent Heart

**FIGURE 3:**
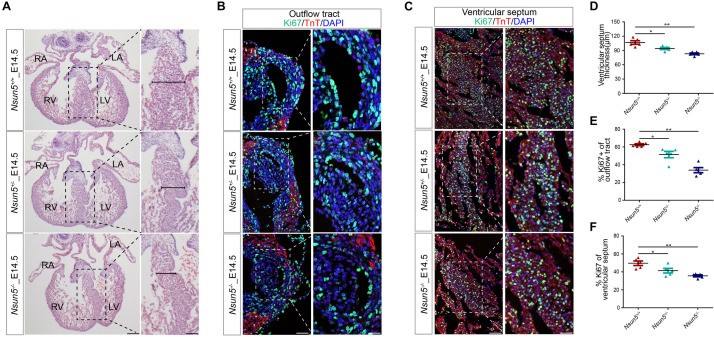
The *Nsun5* deletion reduces cell proliferation. **(A,D)** H&E stained sections from E14.5 hearts indicated a thinner ventricular septum resulting from *Nsun5* deletion. *n* = 5/group. **(B,E)** Quantitative results showed reduced proliferation in *Nsun5* deletion OFT aorta area by immunofluorescence staining. The data was presented as the ratio of Ki67^+^ cells/total OFT aorta area cells. Cardiomyocytes were labeled by TnT. *n* = 5/group. **(C,F)** Quantitative results showed reduced proliferation in *Nsun5* deletion ventricular septum by immunofluorescence staining. The data was presented as the ratio of Ki67^+^ cells/total ventricular septum cells. *n* = 5/group. Scale bar = 50 or 25 μm. All the data were the mean ± SEMs of three dependent experiments. One-way ANOVA test used for statistical calculation. *P < 0.05, **P < 0.01.

Further analysis of E14.5 *Nsun5*^+/–^ *and Nsun5*^–/–^ hearts by H&E staining revealed that the deletion resulted in a thin ventricular septum (Figures 3A,D), indicating a growth defect. We confirmed this by immunofluorescence staining for Ki67, which showed significantly reduced cell proliferation in OFT and the ventricular septum in E14.5 *Nsun5*^+/–^ and *Nsun5*^–/–^ hearts (Figures 3B,C,E,F). We also observed a significantly decreased thickness of the compact myocardium and reduced numbers of Ki67 positive cells in the myocardial wall of E14.5 *Nsun5*^+/–^ *and Nsun5*^–/–^ hearts (Supplementary Figures 6A,B,D,E). These findings indicate cell proliferation defects are present in the *Nsun5* deletion embryo OFT.

### *Nsun5* Regulates *Tpm1* Translation by m^5^C Methyltransferase

**FIGURE 4:**
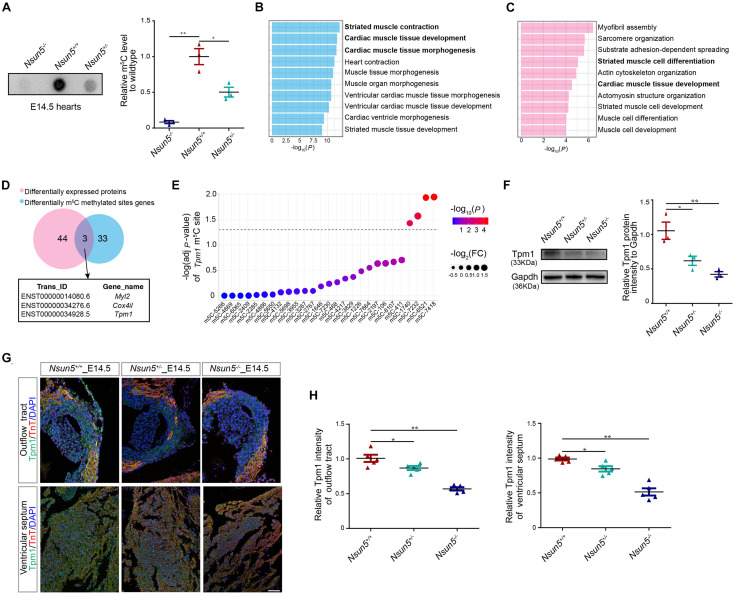
*Nsun5* regulated *Tpm1* as a m^5^C methyltransferase. **(A)** The mRNAs isolated from E14.5 *Nsun5* hearts were used in dot blot analyses with m^5^C antibody. *n* = 3/group. **(B)** GO-BP analysis for the DMSs genes in E14.5 *Nsun5*^+/+^ and *Nsun5*^– /–^ hearts. **(C)** GO-BP analysis for the differentially expressed proteins in E14.5 *Nsun5*^+/+^ and *Nsun5*^– /–^ hearts. **(D)** The overlap of identified DMSs genes and differentially expressed proteins by BS-seq and LC-MS/MS in *Nsun5*^– /–^ E14.5 hearts when compared with *Nsun5*^+/+^ controls. **(E)** DMSs in *Tpm1*. Fold change of each DMS was indicated by the node size. *P-*value of each DMS was indicated by the bar color. The black horizontal line represents *P* = 0.05. **(F)** The protein expression of Tpm1 confirmed by western blot in E14.5 *Nsun5* hearts. *n* = 3/group. **(G,H)** Quantitative results showed reduced Tpm1 intensity in *Nsun5* deletion OFT aorta area and ventricular septum by immunofluorescence staining. The data was presented as the ratio of Tpm1^+^ area intensity/total area intensity. Cardiomyocytes were labeled by TnT. *n* = 5/group. Scale bar = 50 μm. All the data were the mean ± SEMs of three dependent experiments. One-way ANOVA test and hypergeometric test were used for statistical calculation. *P < 0.05, **P < 0.01.

We examined the m^5^C level in E14.5 hearts and different genotypes of mouse embryo fibroblast (MEF) cells by dot blot assay. The results revealed that *Nsun5*^+/–^ and *Nsun5*^–/–^ hearts and MEF cells had significantly decreased m^5^C efficiency (Figure 4A and Supplementary Figure 7). DMS genes and differentially expressed proteins in E14.5 *Nsun5*^+/+^ and *Nsun5*^–/–^ hearts were detected by BS-seq and LC-MS/MS assays, followed by pathway enrichment analysis. These results indicated that DMSs genes were associated with striated muscle contraction, cardiac muscle tissue development, and cardiac muscle tissue morphogenesis pathways (Figure 4B). In addition, differentially expressed genes at the protein level also showed a positive correlation with striated muscle cell differentiation and cardiac muscle tissue development pathways (Figure 4C). In total, 36 transcripts exhibited DMSs and 47 proteins were significantly differentially expressed in E14.5 *Nsun5*^+/+^ and *Nsun5*^–/–^ hearts (Figure 4D). Between them, we found only 3 overlapping genes, *Myl2*, *Cox4i1*, and *Tpm1*, which were enriched in the cardiac muscle tissue development pathway. Several studies reported that *Myl2* was strongly associated with hypertrophic heart failure and myocardial development (Claes et al., 2016; Yuan et al., 2018; Zaleta-Rivera et al., 2019). Western blot assay showed that expression of *Myl2* was gradually decreased from *Nsun5* wildtype to *Nsun5* null hearts (Supplementary Figure 8A). However, no studies have reported an association between *Cox4i1* and heart development and no significant change of *Cox4i1* expression was observed in different genotypes of E14.5 mice hearts (Supplementary Figure 8B). Of note, *Tpm1* is an essential muscle gene highly expressed in cardiac cells (Cardoso-Moreira et al., 2019) required for heart development (McKeown et al., 2014; England et al., 2017). We found that the m^5^C abundance of four DMSs in *Tpm1* were significantly reduced in E14.5 *Nsun5*^–/–^ hearts (Figure 4E), and confirmed that *Tpm1* protein levels were decreased in E14.5 *Nsun5*^+/–^ and *Nsun5*^–/–^ hearts by western blot assay (Figure 4F). Moreover, immunofluorescence staining for Tpm1 revealed significantly reduced protein levels in the OFT and ventricular septum in E14.5 *Nsun5*^+/–^ *and Nsun5*^–/–^ hearts (Figures 4G,H). Decreased Tpm1 protein levels were also observed in the myocardial wall in E14.5 *Nsun5*^+/–^ *and Nsun5*^–/–^ hearts (Supplementary Figures 6C,F). These results suggest that *Nsun5*-mediated m^5^C modification is required to maintain *Tpm1* expression and that *Nsun5*-dependent *Tpm1* expression might result in OFT cell proliferation.

## Discussion

In the study, we identified an association between putative functional mutations in *NSUN5* and a high risk of TOF in Han Chinese. We also showed that the loss of *Nsun5* led to decreased cardiac cell proliferation, which may be associated with delayed OFT development and a global m^5^C level deficiency in *Nsun5* absent mice. Moreover, we suggest that the essential cardiac-related gene *Tpm1* might be a target of *Nsun5* in the developing heart.

The Online Mendelian Inheritance in Man (OMIM) database currently harbors seven genes that are related to the pathogenesis of TOF including *TBX1*, *NKX2-5*, *GATA4*, *ZFPM2*, *GATA6*, *GDF1*, and *JAG1*. The pathogenic mechanism of these genes in TOF has been confirmed in various experiments (Jerome and Papaioannou, 2001; Lindsay et al., 2001; Merscher et al., 2001; Griffin et al., 2010). Although the functions of these genes in heart development are clear, very few TOF patients harbor mutations in these genes. Therefore, the pathogenic genes and mutations in other TOF patients remain to be identified. *NSUN5*, acting as a cytosine-5 RNA methyltransferase, a post-transcriptional modification (Schosserer et al., 2015), is located in the 7q11.23 locus and patients with this locus deletion have congenital heart malformation, including TOF (Yuan, 2017). This suggests that post-transcriptional modification is necessary for heart development and mutations in genes involved in post-transcriptional modification may regulate the expression of genes during cardiac development (Zaidi et al., 2013). However, the function and mechanism of *NSUN5* in congenital heart disease and heart development remain to be defined. Here, we report that four putative functional mutations in *NSUN5* are associated with TOF. The potential pathogenetic mutations were not identified in whole or East Asian populations in the 1000 Genomes Project or in whole or East Asian populations with an allele frequency ≤0.001 according to the Exome Aggregation Consortium Browser, indicating they might be significantly associated with the pathogenesis of TOF. The mutation c.219_221delAAG (p.K65del) has a deleted lysine and a previous study reported an absence of lysine led to protein synthesis defects (de Proenca et al., 2019). We predicted this mutation might be deleterious, which may affect protein synthesis. Further results of *in vitro* experiments indicated that the mutation c.219_221delAAG (p.K65del) decreased *NSUN5* expression and global m^5^C level. The non-sense mutation c.1236_1240delTGCCT (p.CL404fs^∗^5) in the *S*- adenosylmethionine -dependent methyltransferase domain, which is associated with m^5^C modifications in various kinds of RNAs (Schosserer et al., 2015), led to a frameshift translation. The frameshift translation was predicted to impair *NSUN5* functions and it was reported that *NSUN5* deficiency was associated with a reduction in protein synthesis during mammalian development (Heissenberger et al., 2019). We also found that the mutation c.1236_1240delTGCCT (p.CL404fs^∗^5) led to lower *NSUN5* expression and global m^5^C level. However, the other two mutations domains may not functional because *in vitro* assays showed that there was no significant change in *NSUN5* expression and global m^5^C level between wildtypes and mutant forms. These results indicate that the two functional mutant forms (c.219_221delAAG, p.K65del and c.1236_1240delTGCCT, p.CL404fs^∗^5) play a critical role in affecting *NSUN5* expression and its enzyme activity. In our study, all the predicted deleterious *NSUN5* mutations in TOF patients were heterozygous. A recent study reported that a 33% transposition of the great arteries, a type of OFT defect, had more than one candidate gene hit by putative heterozygous deleterious variants and that the cases might have been affected by polygenic inheritance (Liu et al., 2020). Such an inheritance pattern might account for the findings of our genetic analyses of TOF patients. Moreover, our study also showed that the exon mutations in *NSUN5* (c.219_221delAAG, p.K65del and c.1236_1240delTGCCT, p.CL404fs^∗^5) induced a decrease of *NSUN5* mRNA expression which implied an autoregulation of its transcription level. Previously, Zhou et al. (2013) found that exon variants in FUS is directly correlated with increasing deficiencies in it own mRNA and this dynamic regulation describes a novel mechanism of FUS autoregulation. In addition, miRNAs, as post-transcriptional regulators, may also contribute to SF2/ASF autoregulation (Sun et al., 2010). These studies provided a possible theoretical basis for our findings. In our study, the exon mutations in *NSUN5* were verified to affect its enzyme activity and global m^5^C level, indicating that mutations in *NSUN5*, as well as reduced post-transcriptional m^5^C modification, might resulted in a *NSUN5* autoregulation loop. However, further well-designed studies are needed to validate the potential *NSUN5* autoregulation function.

*NSUN5* encodes a nucleolar protein and functions as a cytosine-5 RNA methyltransferase (Schosserer et al., 2015). As its spatiotemporal expression pattern shows, *Nsun5* expression increased gradually during E12.5, E14.5, E16.5, P1 stages of mouse heart development (Supplementary Figure 9). However, there were diverse cell types that contributed to OFT development, we still do not know which specific cell type is affected by *NSUN5*. Further conditional mutagenesis should be conducted to define *NSUN5* functions. In this study, we confirmed causality in the *Nsun5* deletion mouse model by showing that the absence of *Nsun5* delayed OFT development, which are characteristic TOF features in human patients. We provide evidence supporting the idea that defective cell proliferation underlies structural abnormalities. Also, *Nsun5* is widely expressed in other mouse tissues (Supplementary Figure 9), implying *Nsun5* may play an important role in the development of multiple organs. Zhang et al. (2019) reported that a *Nsun5* deletion led to deficits in spatial cognitive abilities in mice through the disrupted development and function of oligodendrocyte precursor cells. Yuan et al. (2019) reported that a deletion of *Nsun5* decreased the length of the sagittal corpus callosum (CC) and the area of the coronal CC in mice. In addition, Chen et al. (2019) established the critical role of *Nsun5* in the development of the cerebral cortex in mice by regulating the radial glial scaffolds of radial glial cells to control the migration of neocortical neurons. These results inferred that *Nsun5* may contribute to other organs development.

Over 100 types of RNA modifications have been reported for human RNAs (Meyer and Jaffrey, 2014; Engel and Chen, 2018). Among them, m^5^C is prevalent in mRNA (Squires et al., 2012; Amort et al., 2017; Yang et al., 2017). Recent studies indicated that m^5^C modified by NSUN family members is involved in developmental disease. Furthermore, the dynamic regulation of m^5^C modified by *Nsun2* occurs during testis development (Yang et al., 2017). In addition, another study revealed that the loss of *Nsun2* reduced m^5^C levels leading to the increased apoptosis of cortical, hippocampal, and striatal neurons, which highlights the critical role of m^5^C modified by *Nsun2* in development (Blanco et al., 2014). Mechanistically, we identified an m^5^C deficiency in *Nsun5* knockout mice, which was reported to drive the global reduction of protein synthesis and altered translational programs that together promote developmental abnormalities (Blanco et al., 2016). Recently, a study concluded that *NSUN5* heterozygosity led to haploinsufficiency and decreased RNA methylation, which regulated target genes expression (Heissenberger et al., 2019). In our study, we identify that the essential cardiac related gene *Tpm1* might be a target of *Nsun5* in the developing heart by examining the overlap between DMS genes and differentially expressed proteins in E14.5 *Nsun5*^+/+^ and *Nsun5*^–/–^ hearts.

*TPM1* is a member of the tropomyosin family of highly conserved actin-binding proteins and is broadly expressed in the developing OFT (Liu X. et al., 2019) (Supplementary Figure 10). Importantly, *TPM1* regulates the expressions of genes involved in cell proliferation and the heart development pathway (Takasaki et al., 2018), and defective cell proliferation is a major contributor to OFT defects during development (Liu J. et al., 2019). *Tpm1* knockdown resulted in OFT defects during cardiac looping and ventricular formation in zebrafish (England et al., 2017). Lacking *Tpm1* mice are embryonic lethal at E9.5 with enlarged, misshapen, and non-beating hearts characterized by an abnormally thin myocardium (McKeown et al., 2014). In our study, *Nsun5*-mediated m^5^C modifications might be positively correlated with the expression of Tpm1 protein levels in E14.5 mouse hearts. Thus, we propose that the *NSUN5*/*TPM1* axis regulates proper OFT development by modifying proliferation. However, further experiments should be conducted to explore the role of *TPM1* during OFT morphogenesis by *Tpm1* conditional OFT deletion mouse model. At present, we cannot rule out the idea that *NSUN5* might affect cardiac development by other biological pathways.

## Conclusion

Our data demonstrate that *NSUN5* may play a critical role in OFT development by post-transcriptional m^5^C modification. This study suggests that *NSUN5* might be a candidate gene for TOF and is the first to indicate that m^5^C modification might play an important role during heart development.

## Data Availability Statement

The datasets generated for this study are available on request to the corresponding authors.

## Ethics Statement

The studies involving human participants were reviewed and approved by The Ethical Committee of Nanjing Medical University. Written informed consent to participate in this study was provided by the participants’ legal guardian/next of kin. The animal study was reviewed and approved by The Institutional Animal Care Committee of Nanjing Medical University. Written informed consent was obtained from the individual(s), and minor(s)’ legal guardian/next of kin, for the publication of any potentially identifiable images or data included in this article.

## Author Contributions

ZH and CD contributed to the conception or design of the work. YWa, TJ, and JX contributed to the analysis and interpretation of data for the work. YG and YZ contributed to the acquisition of data for the work. YWa and TJ drafted the manuscript. YL, YWu, WL, CW, BS, XM, XW, and BZ critically revised themanuscript. All gave final approval and agree to be accountable for all aspects of work ensuring integrity and accuracy.

## Conflict of Interest

The authors declare that the research was conducted in the absence of any commercial or financial relationships that could be construed as a potential conflict of interest.

## Supplementary Material

Supplementary Figure 1 Schematic of work flow for this study. This flow chart shows the process of this research. All the criterions and results with selection of mutations after *NSUN5* coding region direct Sanger sequencing are listed in the figure.

Supplementary Figure 2 All predicted pathogenic mutations detected by direct Sanger sequencing in TOF patients. The panel demonstrates the mutations detected and validated by Sanger sequencing. They are c.219_221delAAG (p.K65del); (c.324G > T, p.A100S); c.1236_1240delTGCCT (p.CL404fs^∗^5); c.1370_1373delAGAA (p.KE448fs^∗^17).

Supplementary Figure 3 Three-dimensional ribbon model of *NSUN5* mutations position. **(A)** Residue at the amino acid position 65 is highlighted as green spheres. **(B)** Residue at the amino acid position 100 is highlighted as green spheres. **(C)** Residue at the amino acid position 404 is highlighted as green spheres. **(D)** Residue at the amino acid position 448 is highlighted as green spheres. All ribbon models were predicted by software SWISS-PDB VIEWER 4.10.

Supplementary Figure 4 Genotype frequency analysis of F_2_ offspring from *Nsun5*^+/–^ heterozygote crosses. A decreased amount of *Nsun5*^–/–^ mice were identified among 153 P1 newborns with an imbalanced Mendelian ratio. The genotype ratio from E14.5 embryos was in accordance with the expected Mendelian ratio. Embryo analysis showed *Nsun5*^–/–^ lethality between E14.5 and P1. Pearson’s Chi-squared test was used for statistical calculation.

Supplementary Figure 5 OFT septation development delay shown in E15.5 and P1 *Nsun5* absent mice. **(A)** Representative H&E staining of E15.5 hearts. *Nsun5*^+/–^ and *Nsun5*^–/–^ mice E15.5 hearts clearly showed OFT aorta misalignment (asterisk) and septation defect (arrow). **(B)** Representative H&E staining of P1 hearts. *Nsun5*^+/–^ and *Nsun5*^–/–^ mice P1 hearts clearly showed OFT aorta misalignment (asterisk) and septation defect (arrow). **(C)** Frequency analysis of OFT development delay in E15.5 embryo hearts. **(D)** Frequency analysis of OFT development delay in P1 embryo hearts. Scale bar = 100 or 50 μm. Pearson’s Chi-squared test was used for statistical calculation.

Supplementary Figure 6 The *Nsun5* deletion reduces cell proliferation in myocardium wall via regulating *Tpm1* expression. **(A,D)** H&E stained sections from E14.5 hearts indicated a thinner myocardium wall resulting from *Nsun5* deletion. *n* = 5/group. **(B,E)** Quantitative results showed reduced proliferation in *Nsun5* deletion myocardium wall by immunofluorescence staining. The data was presented as the ratio of Ki67^+^ cells/total myocardium wall cells. Cardiomyocytes were labeled by TnT. *n* = 5/group. **(C,F)** Quantitative results showed reduced Tpm1 intensity in *Nsun5* deletion myocardium wall by immunofluorescence staining. The data was presented as the ratio of Tpm1^+^ area intensity/total area intensity. Cardiomyocytes were labeled by TnT. *n* = 5/group. Scale bar = 50 or 25 μm. All the data were the mean ± SEMs of three dependent experiments. One-way ANOVA test was used for statistical calculation. *^∗^P* < 0.05, *^∗∗^P* < 0.01.

Supplementary Figure 7 The *Nsun5* deletion in MEF cells reduces global m^5^C level. The mRNAs isolated from *Nsun5* MEF cells were used in dot blot analyses with m^5^C antibody. *n* = 3/group. One-way ANOVA test was used for statistical calculation. *^∗^P* < 0.05, *^∗∗^P* < 0.01.

Supplementary Figure 8 Western blot assay for *Myl2* and *Cox4i1* expression. **(A)** The protein expression of Myl2 confirmed by western blot in E14.5 *Nsun5* hearts. *n* = 3/group. **(B)** The protein expression of Cox4i1 confirmed by western blot in E14.5 *Nsun5* hearts. *n* = 3/group. One-way ANOVA test was used for statistical calculation. *^∗∗^P* < 0.01.

Supplementary Figure 9 The *Nsun5* spatiotemporal expression pattern. **(A)** The *Nsun5* spatiotemporal expression pattern in mRNA level at E12.5 E14.5, E16.5, and P1 with multiple tissues. **(B)** The *Nsun5* spatiotemporal expression pattern in protein level at E12.5 E14.5, E16.5, and P1 with mouse embryo hearts.

Supplementary Figure 10 The *Tpm1* expression in OFT cell types. Various OFT cell types expressed *Tpm1* in public single cell RNA-seq data.

Supplementary Figure 11 Full western blot membranes in the study. **(A)** Full western blot membrane for *NSUN5* expression assay in different mutant forms. **(B)** Full western blot membrane for *Nsun5* spatiotemporal expression in mouse embryo hearts. **(C)** Full western blot membrane for *Nsun5* and *Myl2* expression assay in E14.5 hearts. **(D)** Full western blot membrane for *Tpm1* expression assay in E14.5 hearts. **(E)** Full western blot membrane for *Cox4i1* expression assay in E14.5 hearts.
