# Supplementary figures and images for: Mutations in RNA Methyltransferase Gene NSUN5 Confer High Risk of Outflow Tract Malformation
Source: Front Cell Dev Biol. 2021 Apr 21;9:623394. doi: 10.3389/fcell.2021.623394 (PMC8097101; doi:10.3389/fcell.2021.623394)

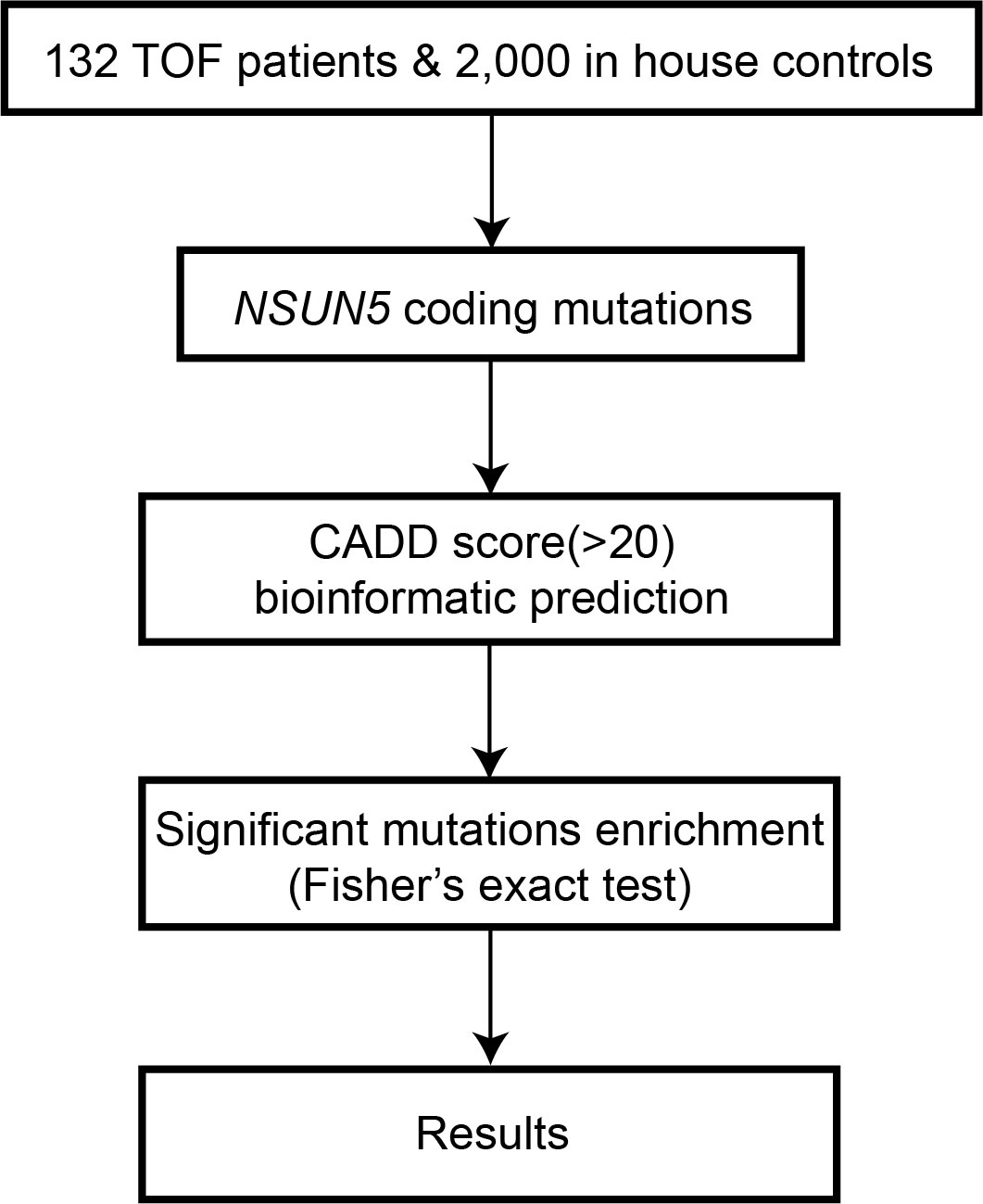

Supplement: Supplementary Figure 1 — Schematic of work flow for this study. This flow chart shows the process of this research. All the criterions and results with selection of mutations after NSUN5 coding region direct Sanger sequencing are listed in the figure. [file Image_1.JPEG]

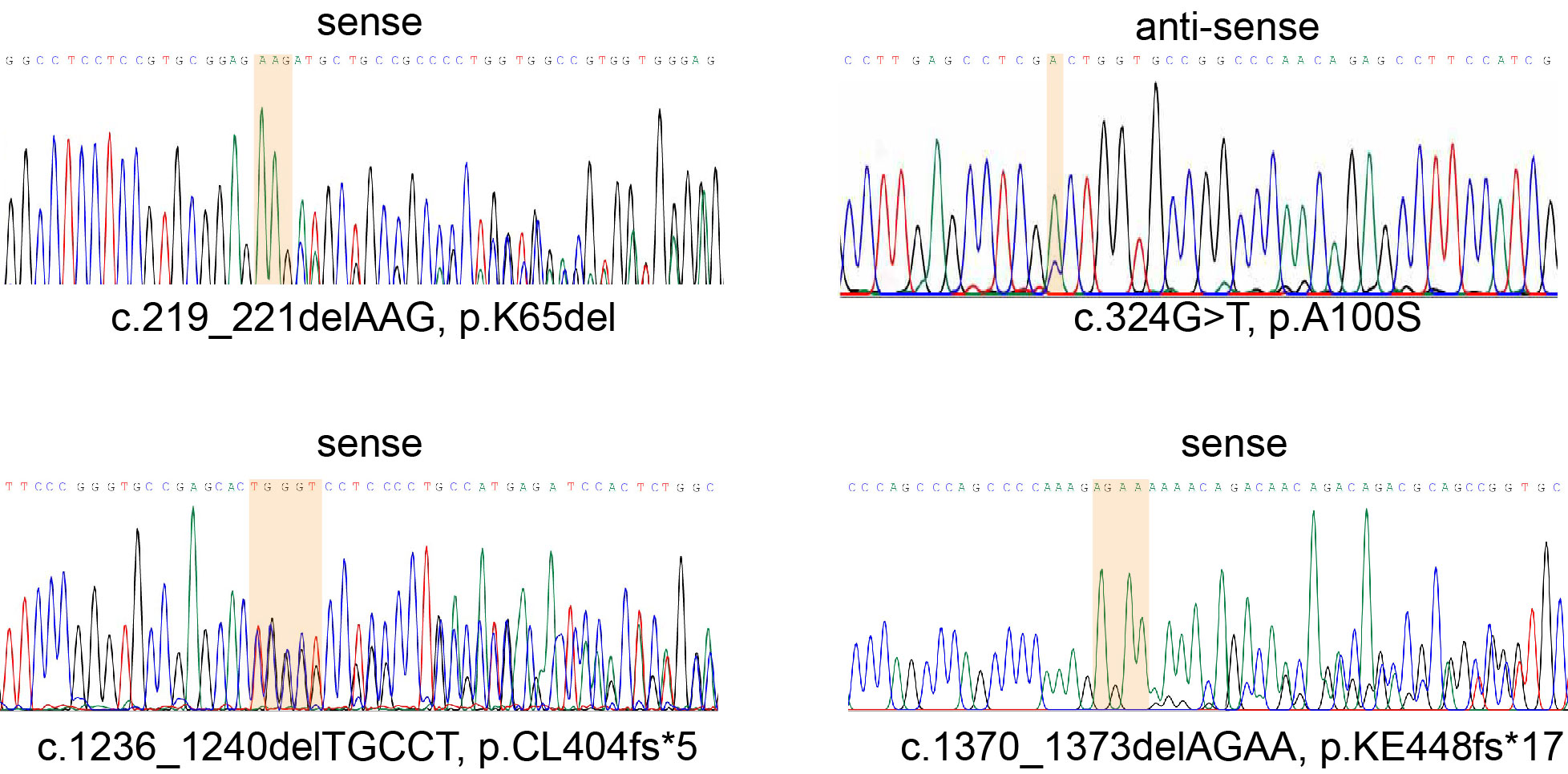

Supplement: Supplementary Figure 2 — All predicted pathogenic mutations detected by direct Sanger sequencing in TOF patients. The panel demonstrates the mutations detected and validated by Sanger sequencing. They are c.219_221delAAG (p.K65del); (c.324G > T, p.A100S); c.1236_1240delTGCCT (p.CL404fs∗5); c.1370_1373delAGAA (p.KE448fs∗17). [file Image_2.JPEG]

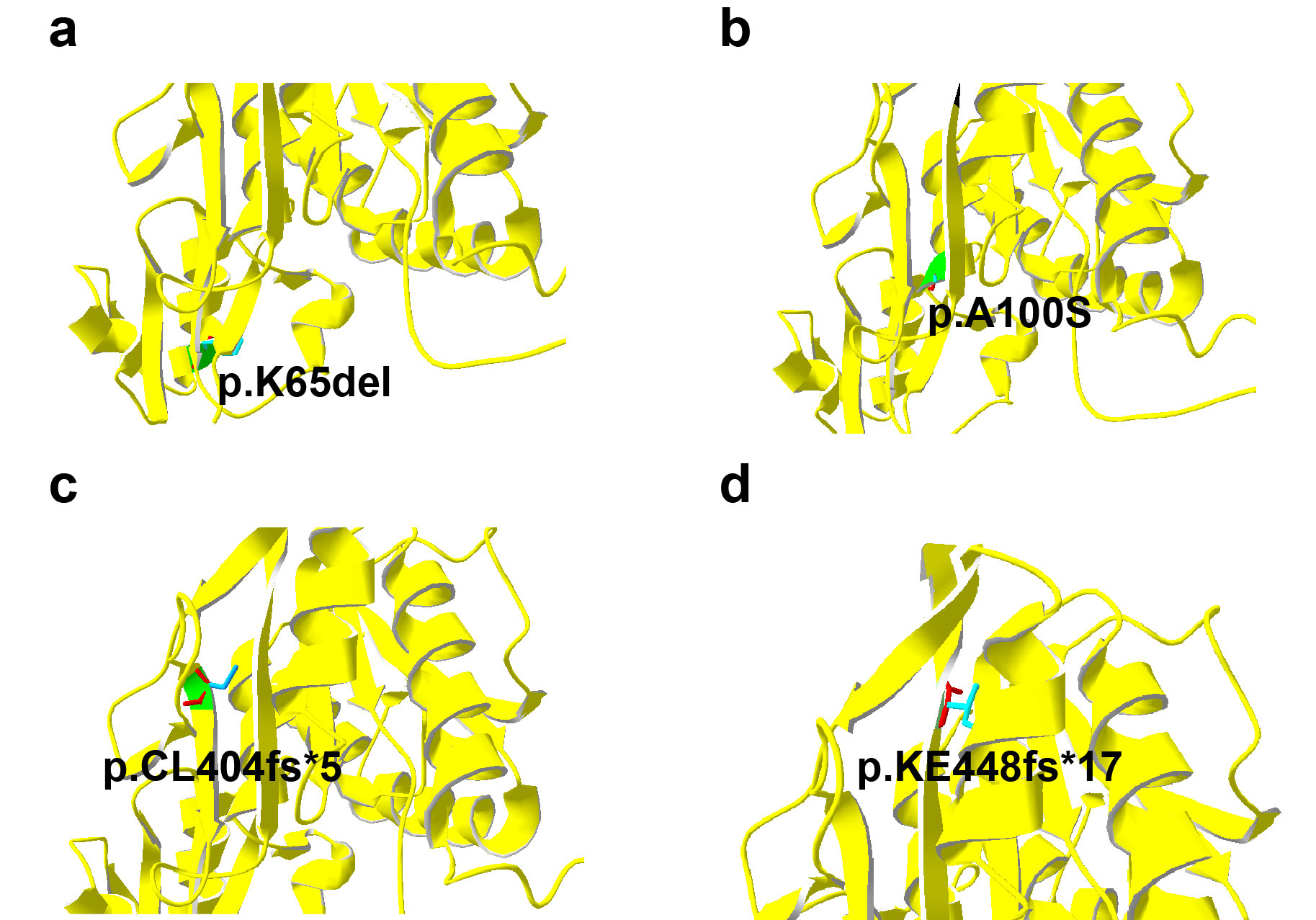

Supplement: Supplementary Figure 3 — Three-dimensional ribbon model of NSUN5 mutations position. (A) Residue at the amino acid position 65 is highlighted as green spheres. (B) Residue at the amino acid position 100 is highlighted as green spheres. (C) Residue at the amino acid position 404 is highlighted as green spheres. (D) Residue at the amino acid position 448 is highlighted as green spheres. All ribbon models were predicted by software SWISS-PDB VIEWER 4.10. [file Image_3.JPEG]

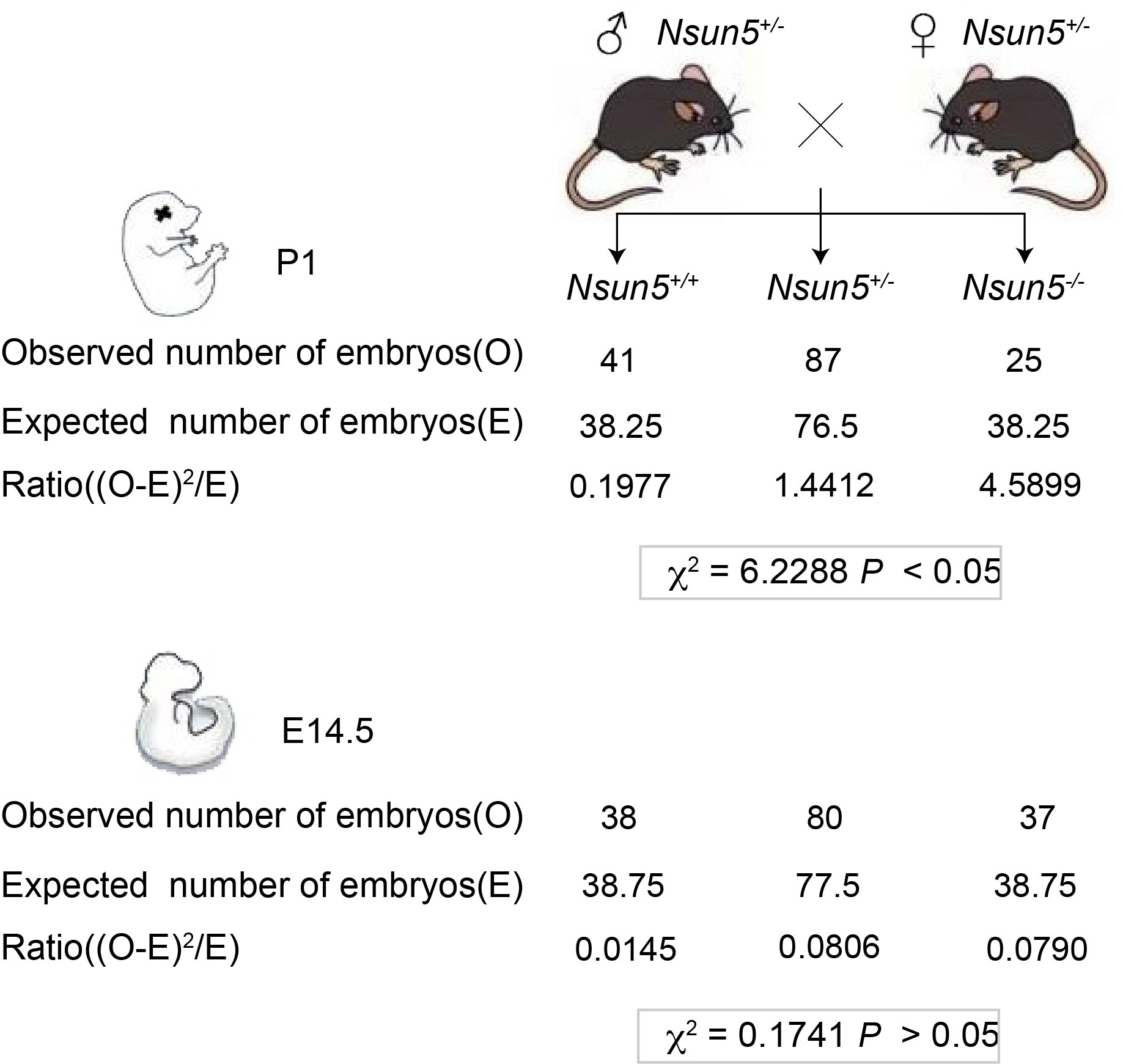

Supplement: Supplementary Figure 4 — Genotype frequency analysis of F2 offspring from Nsun5+/– heterozygote crosses. A decreased amount of Nsun5–/– mice were identified among 153 P1 newborns with an imbalanced Mendelian ratio. The genotype ratio from E14.5 embryos was in accordance with the expected Mendelian ratio. Embryo analysis showed Nsun5–/– lethality between E14.5 and P1. Pearson’s Chi-squared test was used for statistical calculation. [file Image_4.JPEG]

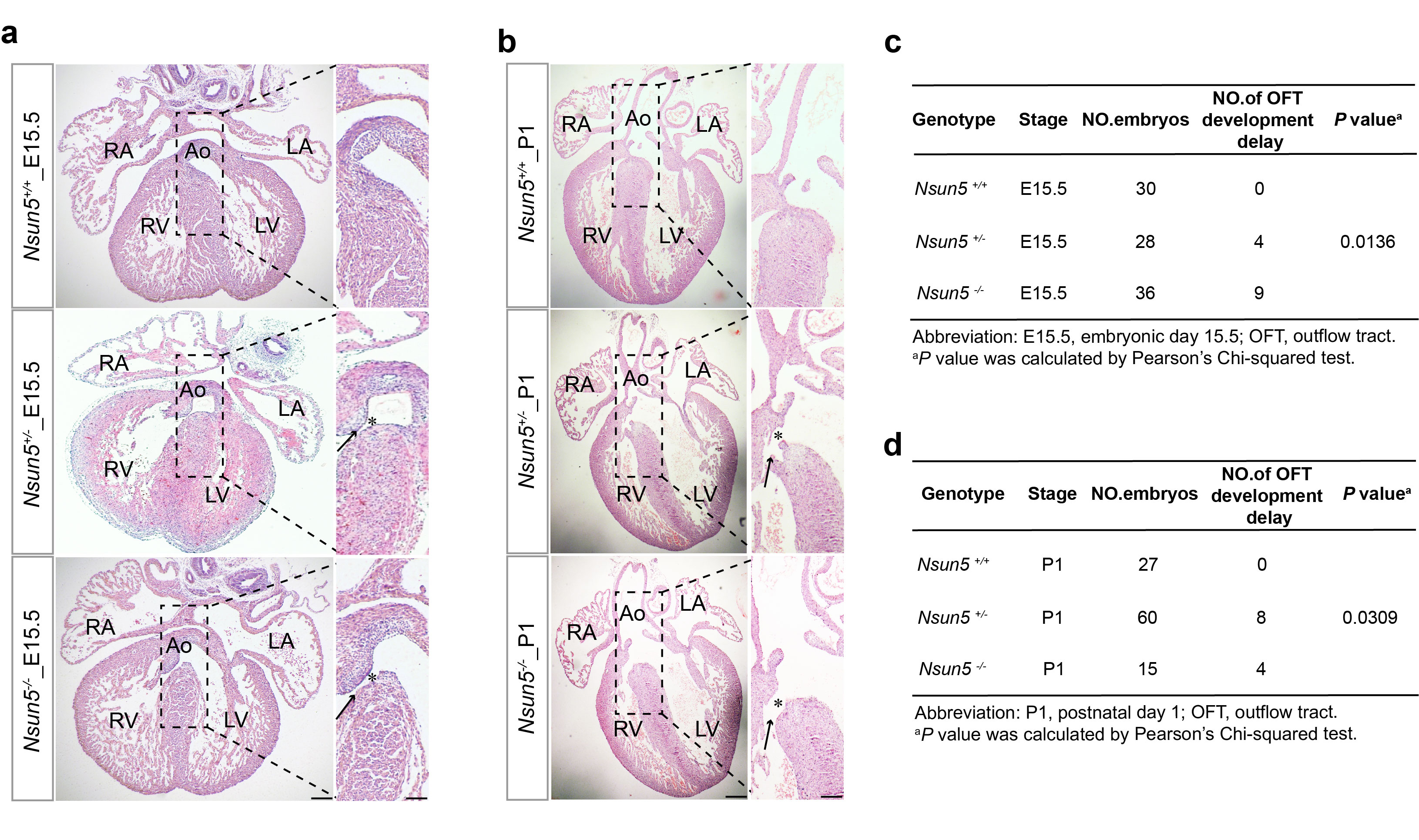

Supplement: Supplementary Figure 5 — OFT septation development delay shown in E15.5 and P1 Nsun5 absent mice. (A) Representative H&E staining of E15.5 hearts. Nsun5+/– and Nsun5–/– mice E15.5 hearts clearly showed OFT aorta misalignment (asterisk) and septation defect (arrow). (B) Representative H&E staining of P1 hearts. Nsun5+/– and Nsun5–/– mice P1 hearts clearly showed OFT aorta misalignment (asterisk) and septation defect (arrow). (C) Frequency analysis of OFT development delay in E15.5 embryo hearts. (D) Frequency analysis of OFT development delay in P1 embryo hearts. Scale bar = 100 or 50 μm. Pearson’s Chi-squared test was used for statistical calculation. [file Image_5.JPEG]

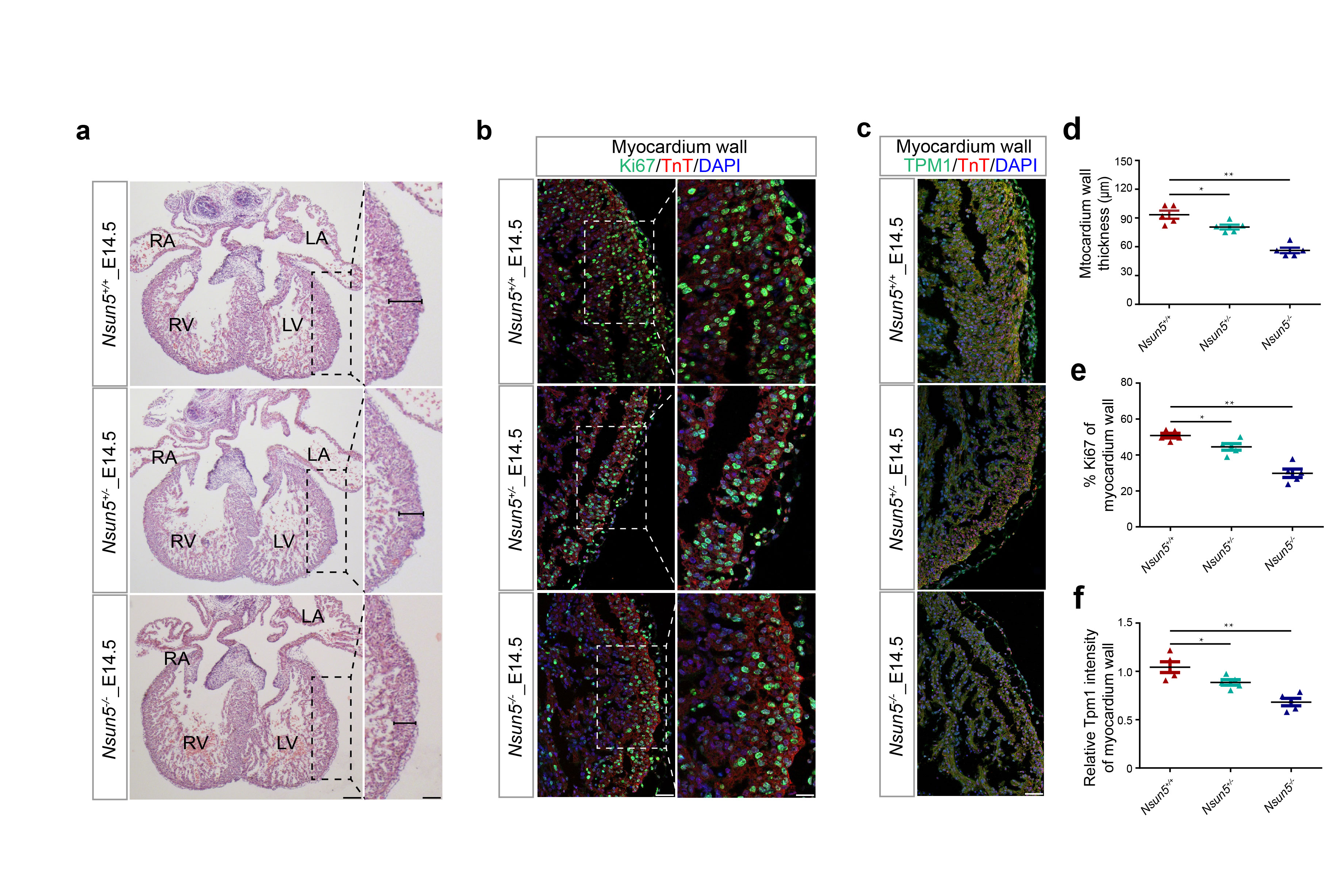

Supplement: Supplementary Figure 6 — The Nsun5 deletion reduces cell proliferation in myocardium wall via regulating Tpm1 expression. (A,D) H&E stained sections from E14.5 hearts indicated a thinner myocardium wall resulting from Nsun5 deletion. n = 5/group. (B,E) Quantitative results showed reduced proliferation in Nsun5 deletion myocardium wall by immunofluorescence staining. The data was presented as the ratio of Ki67+ cells/total myocardium wall cells. Cardiomyocytes were labeled by TnT. n = 5/group. (C,F) Quantitative results showed reduced Tpm1 intensity in Nsun5 deletion myocardium wall by immunofluorescence staining. The data was presented as the ratio of Tpm1+ area intensity/total area intensity. Cardiomyocytes were labeled by TnT. n = 5/group. Scale bar = 50 or 25 μm. All the data were the mean ± SEMs of three dependent experiments. One-way ANOVA test was used for statistical calculation. ∗P < 0.05, ∗∗P < 0.01. [file Image_6.JPEG]

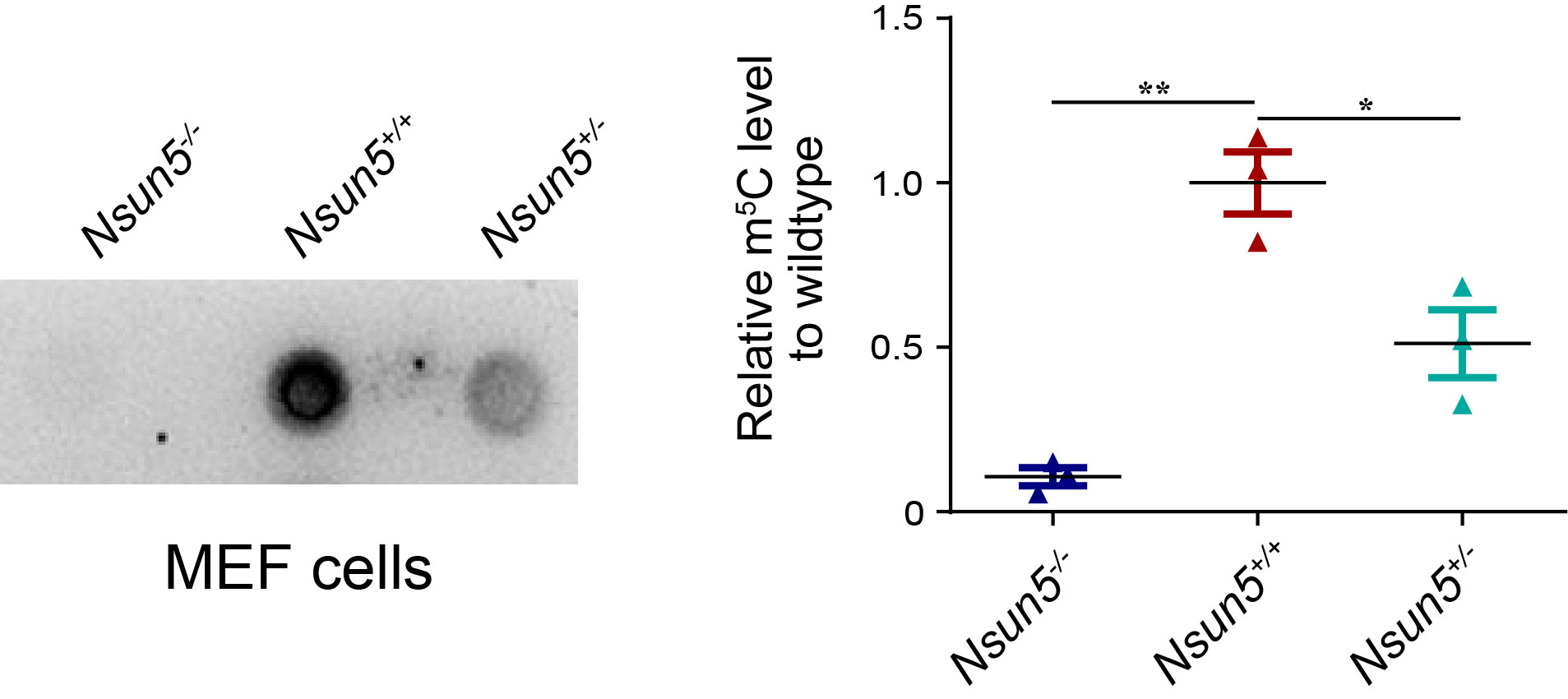

Supplement: Supplementary Figure 7 — The Nsun5 deletion in MEF cells reduces global m5C level. The mRNAs isolated from Nsun5 MEF cells were used in dot blot analyses with m5C antibody. n = 3/group. One-way ANOVA test was used for statistical calculation. ∗P < 0.05, ∗∗P < 0.01. [file Image_7.JPEG]

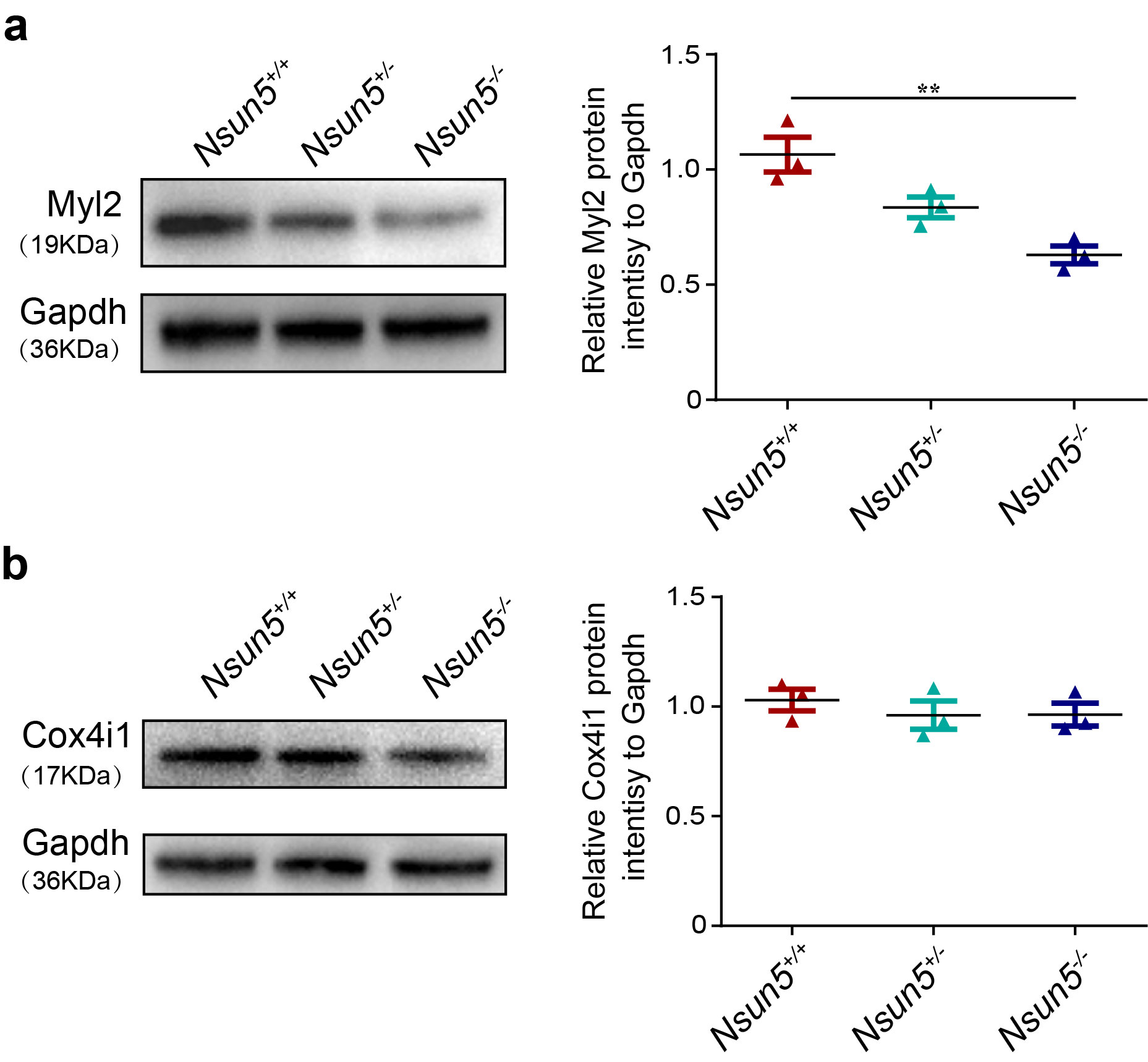

Supplement: Supplementary Figure 8 — Western blot assay for Myl2 and Cox4i1 expression. (A) The protein expression of Myl2 confirmed by western blot in E14.5 Nsun5 hearts. n = 3/group. (B) The protein expression of Cox4i1 confirmed by western blot in E14.5 Nsun5 hearts. n = 3/group. One-way ANOVA test was used for statistical calculation. ∗∗P < 0.01. [file Image_8.JPEG]

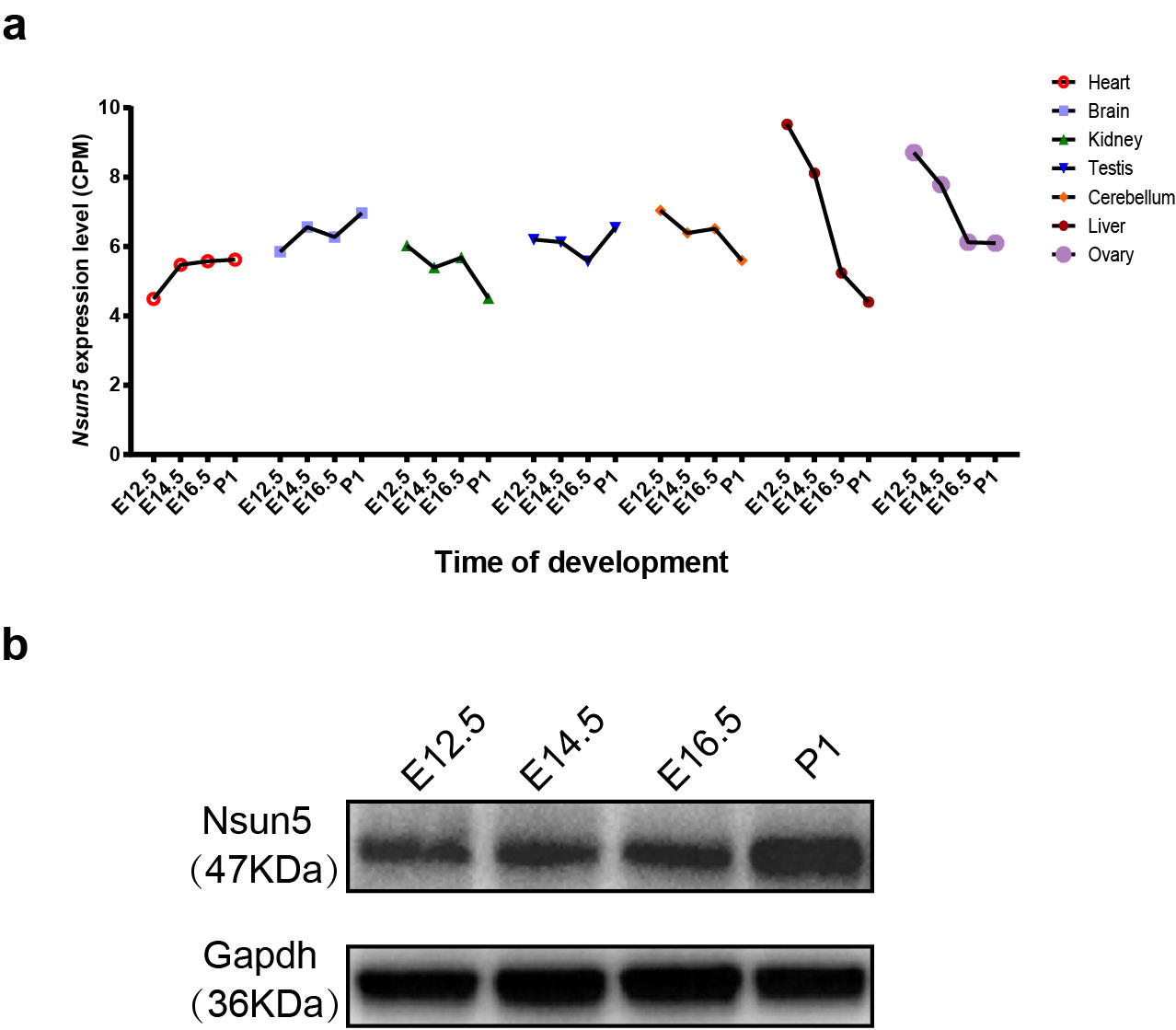

Supplement: Supplementary Figure 9 — The Nsun5 spatiotemporal expression pattern. (A) The Nsun5 spatiotemporal expression pattern in mRNA level at E12.5 E14.5, E16.5, and P1 with multiple tissues. (B) The Nsun5 spatiotemporal expression pattern in protein level at E12.5 E14.5, E16.5, and P1 with mouse embryo hearts. [file Image_9.JPEG]

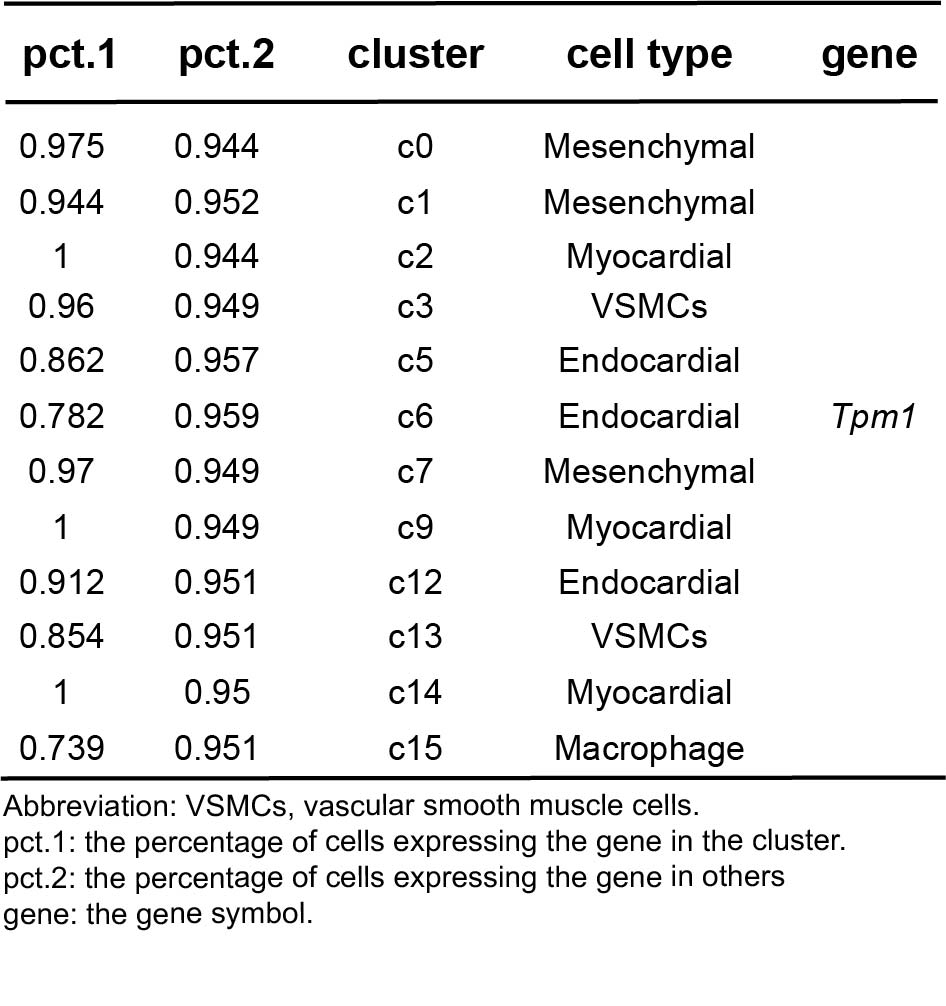

Supplement: Supplementary Figure 10 — The Tpm1 expression in OFT cell types. Various OFT cell types expressed Tpm1 in public single cell RNA-seq data. [file Image_10.JPEG]

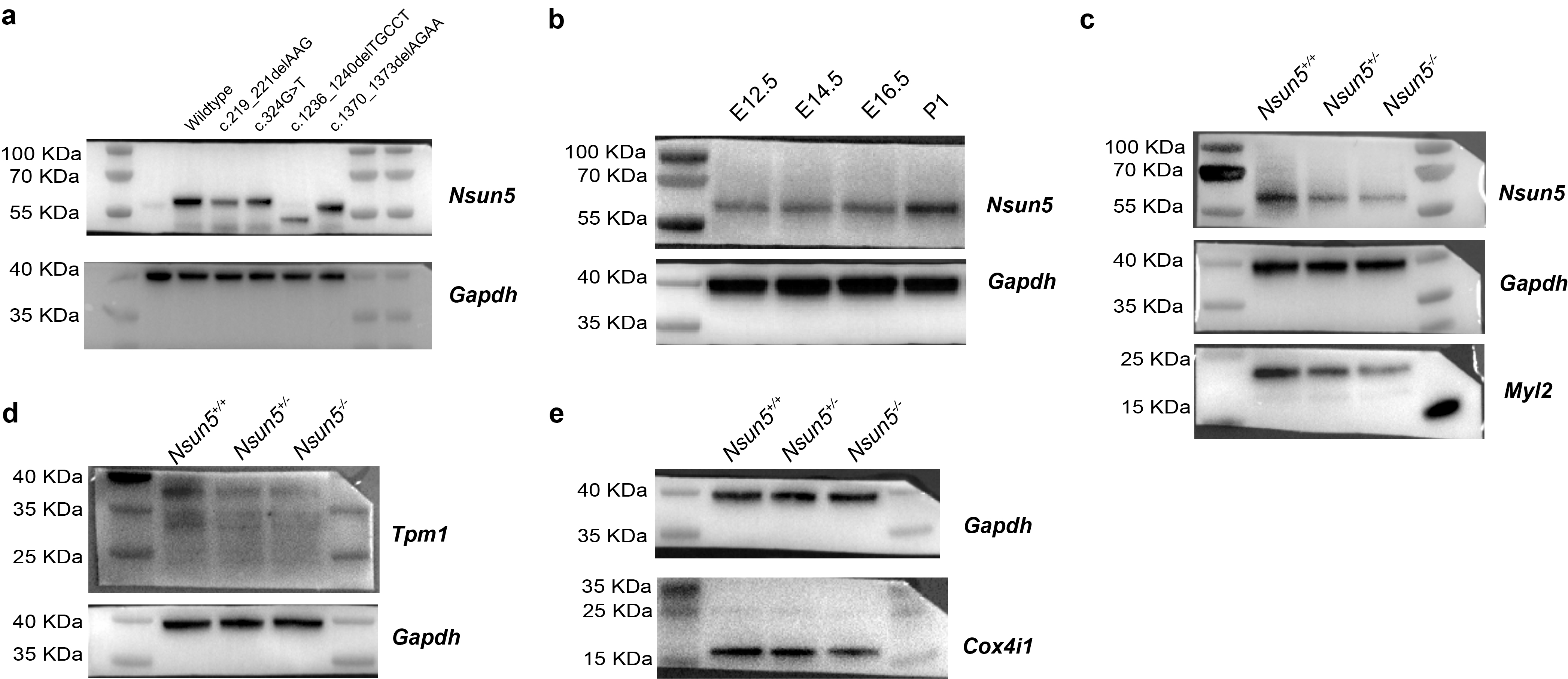

Supplement: Supplementary Figure 11 — Full western blot membranes in the study. (A) Full western blot membrane for NSUN5 expression assay in different mutant forms. (B) Full western blot membrane for Nsun5 spatiotemporal expression in mouse embryo hearts. (C) Full western blot membrane for Nsun5 and Myl2 expression assay in E14.5 hearts. (D) Full western blot membrane for Tpm1 expression assay in E14.5 hearts. (E) Full western blot membrane for Cox4i1 expression assay in E14.5 hearts. [file Image_11.JPEG]
