# Supplementary material for: Mutations in RNA Methyltransferase Gene NSUN5 Confer High Risk of Outflow Tract Malformation
Source: Front Cell Dev Biol. 2021 Apr 21;9:623394. doi: 10.3389/fcell.2021.623394 (PMC8097101; doi:10.3389/fcell.2021.623394)
Supplement: Supplementary file 12 [file Table_1.DOCX]

**Supplementary Table 1. PCR primers used for Sanger sequencing.**

| **Primer** | **Sequence(5'>3')** | **Length(bp)** |
| --- | --- | --- |
| *NSUN5*_Exon1-F | CTGCTTCACGTTCTGTGTGG | 335 |
| *NSUN5*_Exon1-R | GAAGGTCGTGCTTTTTGACC |  |
| *NSUN5*_Exon2-F | GGAAGGTCGGCTCATACTC | 410 |
| *NSUN5*_Exon2-R | AGCAACTTCCAGGTAGCGGA |  |
| *NSUN5*_Exon3-F | TGAGCAGTGAGTAGGCAGGA | 290 |
| *NSUN5*_Exon3-R | GCTTCATGCTTAGGGCATTT |  |
| *NSUN5*_Exon4-F | TCTTCCCAATACCCCAGACA | 203 |
| *NSUN5*_Exon4-R | TCCTGCCTACTCACTGCTCA |  |
| *NSUN5*_Exon5-F | TCAGAAGAGCAGCCAAGTGA | 476 |
| *NSUN5*_Exon5-R | ATTCCGCCCATCTCTAGCTT |  |
| *NSUN5*_Exon6-F | CCCACTGAAAGGCACAGAAT | 244 |
| *NSUN5*_Exon6-R | GAATGTGTAGGCACGGGAAG |  |
| *NSUN5*_Exon7-F | CCCAAGCCCATTAGTGTCAG | 352 |
| *NSUN5*_Exon7-R | CATGTTCTGGTTGGCTGTGA |  |
| *NSUN5*_Exon8-F | GGTAAGAGAGCAGCTCACC | 310 |
| *NSUN5*_Exon8-R | AGCCTGTGAAGCTATGTAA |  |
| *NSUN5*_Exon9-F | GTTCTGGTGCTGATGCTTTG | 391 |
| *NSUN5*_Exon9-R | CCAGGAGGAGAATGAAGACG |  |
| *NSUN5*_Exon10-F | GGCTGGGACTTTCCATTACA | 543 |
| *NSUN5*_Exon10-R | CTCAGCAGTGGCTTCTTCGT |  |

**Supplementary Table 2. sgRNAs and primers of CRISPR/Cas9-mediated gene editing and quantitative real-time PCR (qRT-PCR) assay.**

| ***Nsun5*** |  | **Sequence(5'>3')** |
| --- | --- | --- |
| sgRNA_Exon3 | For_1 | TAGGCCCAGCAGAGCCTTCCAT |
|  | Rev_1 | AAACATGGAAGGCTCTGCTGGG |
|  | For_2 | TAGGCTGAGCTGGCCCGACTCA |
|  | Rev_2 | AAACTGAGTCGGGCCAGCTCAG |
| Genotype_Primers | F | CTGTCCAGGTGCTAGTGTATG |
|  | R | GGTCCTCATTTCGGCTCAC |
| qRT-PCR_Pimers | F | ATTTTCTCCTGGACCCCTTG |
|  | R | ATCCAGGTCAAAGGCAAAGA |

**Supplementary Table 3. All *NSUN5* coding mutations identified in TOF patients.**

| **Gene^a^** | **Variant**  **coordinates^b^** | **cDNA mutation** | **Protein alteration** | **MAF_case** | **AF_in-house control** | **CADD score^c^** |
| --- | --- | --- | --- | --- | --- | --- |
| *NSUN5* | Chr7: 72722805 | c.9C>T | N/A | 1/264 | 7/4000 | 18.79 |
| ***NSUN5*** | **Chr7: 72722451** | **c.219_221 delAAG** | **p.K65del** | **1/264** | **0/4000** | **22.1** |
| ***NSUN5*** | **Chr7: 72721673** | **c.324G>T** | **p.A100S** | **1/264** | **0/4000** | **21.1** |
| *NSUN5* | Chr7: 72718288 | c.899G>T | p.P291 | 2/264 | 4/4000 | 4.903 |
| *NSUN5* | Chr7: 72718278 | c.909G>A | p.R295C | 8/264 | 66/4000 | 11.82 |
| *NSUN5* | Chr7: 72718026 | c.968C>G | p.P314 | 1/264 | 3/4000 | 7.277 |
| *NSUN5* | Chr7: 72717933 | c.1061C>T | p.A345 | 76/264 | 474/4000 | 5.432 |
| *NSUN5* | Chr7: 72717888 | c.1106G>C | p.S360 | 1/264 | 0/4000 | 11.65 |
| ***NSUN5*** | **Chr7: 72717673** | **c.1236_1240delTGCCT** | **p.CL404fs*5** | **1/264** | **0/4000** | **32.0** |
| ***NSUN5*** | **Chr7:72717464** | **c.1370_1373delAGAA** | **p.KE448fs*17** | **1/264** | **0/4000** | **26.6** |
| *NSUN5* | Chr7:72717439 | c.1395G>A | p.R457W | 4/264 | 19/4000 | 9.449 |

Abbreviation: MAF, minor allele frequency; AF, allele frequency.

^a^The accession number for ***NSUN5*** is GenBank: NM_001168347.2;

^b^The ***NSUN5*** gene coding region includes sequence coding for amino acids in protein (CDS) region, untranslated region (UTR) and splicing region;

^c^The function of mutations are predicted by Combined Annotation Dependent Deletion (CADD) tool;

N_case_=132; N_in-house control_=2,000.
